# Evolutionary potential of marine phytoplankton under ocean acidification

**DOI:** 10.1111/eva.12120

**Published:** 2013-11-25

**Authors:** Sinéad Collins, Björn Rost, Tatiana A Rynearson

**Affiliations:** 1 Ashworth Laboratories, Institute of Evolutionary Biology, School of Biological Sciences, University of EdinburghEdinburgh, UK; 2 Alfred Wegener Institute for Polar and Marine ResearchBremerhaven, Germany; 3 Graduate School of Oceanography, University of Rhode IslandNarragansett, RI, USA

**Keywords:** adaptation, experimental evolution, global change, ocean acidification, phytoplankton

## Abstract

Marine phytoplankton have many obvious characters, such as rapid cell division rates and large population sizes, that give them the capacity to evolve in response to global change on timescales of weeks, months or decades. However, few studies directly investigate if this adaptive potential is likely to be realized. Because of this, evidence of to whether and how marine phytoplankton may evolve in response to global change is sparse. Here, we review studies that help predict evolutionary responses to global change in marine phytoplankton. We find limited support from experimental evolution that some taxa of marine phytoplankton may adapt to ocean acidification, and strong indications from studies of variation and structure in natural populations that selection on standing genetic variation is likely. Furthermore, we highlight the large body of literature on plastic responses to ocean acidification available, and evolutionary theory that may be used to link plastic and evolutionary responses. Because of the taxonomic breadth spanned by marine phytoplankton, and the diversity of roles they fill in ocean ecosystems and biogeochemical cycles, we stress the necessity of treating taxa or functional groups individually.

## Introduction

Marine phytoplankton are the fascinating and diverse foundation of the world's largest ecosystem, and their responses to global change will affect marine food webs and global nutrient cycles. These single-celled primary producers drift with tides and currents, are responsible for about half of global carbon fixation, and form the basis of the biological carbon pump that exports fixed carbon to the deep ocean (Falkowski et al. 1998; Field et al. 1998). Due to their pivotal role in ecosystem functioning and biogeochemistry, phytoplankton have been the focus of global change research in marine ecosystems.

Since phytoplankton can divide asexually on the order of hours to days, and can be studied in both natural and laboratory environments over timescales where populations evolve, they present a unique opportunity to quantify evolutionary and plastic responses of populations to global change. However, in contrast to the bulk of studies on this topic, studies using marine phytoplankton tend to focus on determining the evolutionary potential of populations, rather than inferring whether current populations have evolved in response to global change already. Similarly, the majority of reviews in this issue focus on natural populations of multicellular organisms, where disentangling plastic and evolutionary responses is challenging (see Merilä and Hendry, this issue). In contrast, studies using single-celled phytoplankton can make extensive use of experimental evolution, which is a laboratory-based method with the advantage of clearly separating plastic and evolutionary responses to environmental changes, at least for single species in laboratory settings. The challenge with marine phytoplankton thus becomes applying what is learned about evolutionary potential in the laboratory to predicting evolutionary potential in natural populations. While surveys of genetic and phenotypic variation in natural populations of marine phytoplankton exist, and are reviewed here, our ability to interpret them is limited by knowledge of their basic biology, including estimates of gene flow, population size and recombination rates. Finally, studies usually focus on how phenotypic change in globally distributed marine phytoplankton will affect biogeochemical cycles or marine food webs, and so they are mainly concerned with trait evolution rather than population persistence. Here, we first provide background on global change in oceans, and then review studies aimed at understanding how marine phytoplankton communities may evolve in response to global change.

### Impacts of anthropogenic CO_2_ emissions on marine waters

Anthropogenic CO_2_ emissions have various simultaneous effects on marine waters, many of which may be important bottom-up drivers of phytoplankton productivity. Concentrations of CO_2_ and bicarbonate in ocean waters are increasing due to anthropogenic CO_2_ emissions, which has led to a decrease in ocean pH, a phenomenon known as ‘ocean acidification’ (Wolf-Gladrow et al. 1999; Caldeira and Wickett 2003). Since the industrial revolution, the mean pH of the surface ocean has declined by about 0.1 pH units and is expected to decrease an additional 0.3 units by the end of this century, which represents an increase in acidity of approximately 150% (Feely et al. 2009). In addition, average surface water temperatures have already increased by 0.7°C and may rise an additional 3°C by the end of this century. This warming increases stratification of the surface ocean, which in turn alters the light regime in the shallower upper mixed layer and reduces the nutrient supply from below (Rost et al. 2008; Steinacher et al. 2010; Winder and Sommer 2012).

The increase in CO_2_ in the oceans and atmosphere is expected to have numerous effects on marine ecosystems. For example, ocean acidification has the potential to affect phytoplankton community composition (Tortell et al. 2008; Beaufort et al. 2011; Hoppe et al. 2013) and drive physiological and evolutionary change in their constituent species (Lohbeck et al. 2012a), which in turn could affect oxygen production, efficacy of the biological carbon pump and air-water CO_2_ exchange (Rost and Riebesell 2004; Riebesell et al. 2007; Hofmann and Schellnhuber 2009). At the same time, interactions between species, such as grazing intensity and viral infection, are likely to be affected by ocean acidification as well, although little is known about how these and other top-down drivers are expected to change (reviewed by Caron and Hutchins (2013)).

### Marine phytoplankton and global biogeochemical cycles

**Figure 1:**
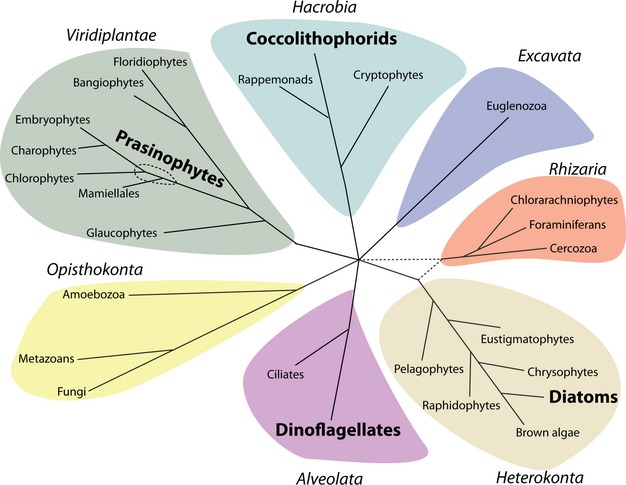
Phylogenetic diversity of eukaryotes. The four major lineages of eukaryotic phytoplankton are highlighted in bold typeface (prasinophytes, coccolithophorids, diatoms and dinoflagellates). These lineages are deeply divergent, highlighting their potentially divergent responses to the effects of climate change. Branching order among some lineages is unresolved (dotted lines).

The enormous impact that marine phytoplankton have on global biogeochemical cycles and marine ecosystem functioning largely motivates which aspects of their ecology, physiology and evolution are routinely measured. Often, their diversity is divided into functional groups based on metabolic capacity and impact on biogeochemical cycles (DeLong 2009; Fuhrman 2009). The main functional groups of marine phytoplankton considered are usually nitrogen fixers (cyanobacteria), silicifiers (diatoms) and calcifiers (coccolithophores). The diversity of roles that marine phytoplankton play in biogeochemical cycles partially explains why particular species are studied, but also highlights their taxonomic diversity – which spans several phyla, and suggests that lumping ‘marine phytoplankton’ into a single group is likely to be misleading (Fig. 1).

Phytoplankton form the base of marine food webs and comprised a taxonomically broad range of organisms that includes both prokaryotes and eukaryotes and consists of thousands of species (Falkowski and Raven 2007). The relative contribution of different taxa to primary production influences the export of organic carbon to the deep ocean, termed the ‘biological pump’ (Volk and Hoffert 1985; De La Rocha and Passow 2007). Different taxa differ in size and composition of their cell walls and coverings, which influence their sinking rates and thus their impact on the biological pump. For example, the smallest constituents of the marine phytoplankton are the picoplankton (0.2–2 μm), which include taxa such as cyanobacteria and prasinophytes, and sink slowly, at rates of < 0.5 m/day (Bach et al. 2012). In contrast, larger cells such as diatoms are covered in biogenic silica, which acts as ballast, and accelerates their sinking to rates of up to 35 m/day (Miklasz and Denny 2010). Coccolithophores are commonly covered with calcium carbonate plates called ‘coccoliths’, which also acts as a ballasting agent, yielding sinking rates of nearly 5 m/day (Bach et al. 2012). In addition to ballast material, processes such as aggregation and the packaging of small cells into large faecal pellets by grazers also affects sinking rates and the efficiency of the biological pump (Klaas and Archer 2002; De La Rocha and Passow 2007).

Given their phylogenetic breadth, different phytoplankton taxa will likely have different responses to climate change. For example, it has been suggested that decreased ocean pH may hinder the ability of coccolithophores to generate calcareous plates, potentially influencing their impact on the biological pump (Riebesell et al. 2000; Langer et al. 2009; Hoppe et al. 2011). Though tiny, coccolithophores are numerous enough for this to matter, with blooms of the cosmopolitan species *Emiliania huxleyi* exceeding 250,000 km^2^. When these cells die and release their coccoliths, the remains of these blooms can be seen from space (Holligan et al. 1993; Balch et al. 1996; Tyrrell and Merico 2004). Because reduced calcification is thought to affect fitness in calcifying coccolithophores (Beaufort et al. 2011), there has been particular interest in the possibility that adaptive evolution may mitigate the reduction in calcification as oceans acidify. There is evidence that this can sometimes occur, since well-calcified morphotypes of coccolithophore*s* have been found in low pH regions of the ocean, despite a general trend of decreasing calcification with rising pCO_2_ levels, though part of this indicates species succession rather than evolution within species (Beaufort et al. 2011).

Because the biological pump largely determines the ability of the ocean to absorb anthropogenic CO_2_ emissions (Maier-Reimer et al. 1996; Doney et al. 2009), both changes to the relative abundance of different functional groups and phenotypic shifts within them, either from plastic responses (i.e. physiological changes) or evolution (i.e. genetic changes) must be considered to understand how phytoplankton responses to ocean acidification may impact global biogeochemical cycles. By virtue of being microbes, marine phytoplankton share some characters that affect their overall ability to evolve in response to environmental change, such as large population sizes and rapid division rates when they are growing. Despite these commonalities, functional groups of marine phytoplankton are not closely related (Fig. 1), and are thus likely to have diverse historical, genetic and physiological constraints on their ability to evolve in a changing ocean, leading to differences in the extent of evolutionary responses and the identity of the traits that evolve.

### Life history and demography

Alongside the wide taxonomic breadth they span, marine phytoplankton differ in several key traits that should affect their evolutionary responses to global change. For example, some marine phytoplankton have a ‘bloom-and-bust’ life history strategy, where they divide rapidly and asexually until they reach high densities (up to millions of cells per litre) over a few days to weeks (Smayda 1997). Some blooms have a characteristic succession of species, so that both direct effects (physiological or evolutionary responses of individual species to acidification) and indirect effects (changes in interactions between species) of global change have the potential to act as evolutionary drivers in blooming phytoplankton. Blooms can end due to grazing (Nejstgaard et al. 1997), nutrient depletion (Smetacek 1985) and viral attack (Bratbak et al. 1990), all of which have the potential to change under ocean acidification.

Bloom-and-bust life histories can include ploidy changes (Frada et al. 2008) and sexual reproduction (Crawford 1995), which makes it challenging to design experiments that incorporate the possibility that life history strategies might evolve in response to ocean acidification, especially since our knowledge of these strategies is limited even for the best-studied phytoplankton. Logistics partly dictate limits to experiments: marine phytoplankton can only be reliably cultured during the asexual phase of their life cycle, so that evolutionary changes in other life history stages are not investigated. In field populations, the extent to which phytoplankton can or do increase variation through sex is also uncertain. For example, estimates of sexual reproduction events range from every 2 to 40 years for diatoms (Mann 1988; Jewson 1992; Holtermann et al. 2010; von Dassow and Montresor 2011). Similarly, rates of dispersal between populations are also unknown. However, isolates of species sampled from different locations or from genetically distinct populations showed heritable phenotypic differentiation (Rynearson and Armbrust 2004; Schaum et al. 2013), suggesting that populations are at least isolated enough to adapt locally. Finally, marine viruses have the potential to move genetic material between populations, and may determine niche specificity in some cases. For example, *Ostreococcus*, a widely distributed picoplankton genus, harbours a large (190 kb) virus encoding several genes with homology to host genes (Weynberg et al. 2009), which has led to the suggestion that light niche is partly determined by the virus (Weynberg et al. 2011). Viruses are important for generating phenotypic diversity, either by encoding phenotypes directly or through host–parasite evolution, in all systems. In marine systems, however, they may be of particular importance if they substantially increase genetic diversity and phenotypic divergence (Rohwer et al. 2009).

Here, we examine how published studies measure or predict responses of marine phytoplankton to climate change. First, we discuss what experimental evolution has revealed about how individual species of marine phytoplankton can evolve in response to ocean acidification. Second, we discuss how detailed studies of physiological (plastic) responses to ocean acidification may help us understand how natural selection may act on marine phytoplankton under global change. Finally, we discuss how much scope there is for evolution in natural populations based on measures of genetic variation in natural populations.

## Experimental evolution

The power of microbial experimental evolution lies in being able to subject many replicate populations to defined environmental or demographic changes for dozens to thousands of generations, and then comparing the evolved populations with either their own ancestor, or with an evolving control population kept in a control environment (Collins 2011b). Experimental evolution allows evolutionary responses to specific environmental changes to be measured directly and quantified in terms of fitness gain and changes in traits of interest, usually photosynthesis-related traits (oxygen evolution or carbon consumption), and cell size or chemical composition (particulate organic carbon, particulate organic nitrogen) (Lohbeck et al. 2012a). In addition, the genetic underpinnings of adaptive change can be investigated (Benner et al. 2013). Recently, oceanographers have begun to adopt experimental evolution methods to measure directly whether marine phytoplankton can evolve over a few hundred generations in response to specific environmental drivers, usually increases in pCO_2_ (see Table 1 and references therein). These studies can be directly compared with studies in freshwater communities (Low-Décarie et al. 2011).

**Table 1:** Summary table comparing plastic and direct evolutionary responses to elevated pCO_2_. In all cases, responses are measured in the same study. Plastic responses are calculated as (growth rate or cell doublings at high pCO_2_) / (growth rate or cell doublings under control conditions) for populations evolved under control conditions, unless otherwise noted. Control conditions are between 380 and 450 ppm pCO_2_ in the studies cited. The evolutionary response is the direct evolutionary response to selection, calculated as (growth at high pCO_2_ of populations selected at high pCO_2_) / (growth at high pCO_2_of populations selected under control conditions), unless otherwise noted. Because evolutionary responses are measured using a fitness proxy (growth rate), plasticity in fitness, rather than other traits, is reported here so that the plastic and evolutionary responses can be meaningfully compared. For both plastic and evolutionary responses, a value of 1 indicates no response, a value <1 indicates a negative response and a value of >1 indicates a positive response. In all cases, values are averages, and should be taken as rough calculations to show sign and approximate magnitude. Since studies use different methods, levels of replication, and measures of growth, meaningful error bars are not possible. All responses were measured at the population level

Organism	Taxon	Environmental change	Plastic response (growth relative to control environment)	Evolutionary response (growth rate relative to control)	Length of evolution experiment (generations)	Other traits measured	Direct evolutionary responses (other traits)	References
*Emiliania huxleyi*	Calcifying coccolithophore	High pCO2 (1100 µatm and 2200 µatm)	0.82	1.02 (ns, selection at 1100 ppm CO_2_); 1.07 (selection at 2200 ppm CO_2_) for populations with initial standing genetic variation	500	PIC, POC, cell size	Generally opposite direction from plastic response	Lohbeck et al. (2012a,b)
*Geophyrocapsa oceanica* (*non-calcifying strain*)	Non-calcifying coccolithophore	High pCO2 (1000 µatm), reduced pH (7.8)	0.9	1.11	670	Photosynthetic carbon fixation, POC, PON, chla	Generally opposite direction from plastic response; no direct response in chla	Jin et al. (2013)
*Ostreococcus tauri*	Chlorophyte (picoplankton)	High pCO2 (1000 ppm constant and 1000 ppm fluctuating)	1.35	0.85 (selection in constant high CO_2_); 0.65 (selection in fluctating high CO_2_)	370–400	Photosynthetic O2 evolution and consumption, cell size, chla, lipid content, POC, PON, TEP	Generally opposite direction from plastic response for O2 evolution and consumption; no direct response in other traits	E. Schaum, S. Collins (in prep)
*Thalassiosira pseudonana*	Diatom	760 µatm pCO2, fluctuations in pH due to culturing method	1.06	0.87	100	Photosynthetic efficiency, POC, PON, expression of CA and Rubisco genes	No	Crawfurd et al. (2011)
*Lingulodinium polydrum*	Dinoflagellate	765 µatm pCO2	7	0.2	48–62	n/a	n/a	Tatters et al. (2013a,b)
*Prorocentrum micans*	Dinoflagellate		1	0.6	58–71			
*Alexandrium sp*.	Dinoflagellate		1	1	34–38			
*Gonyaulax sp*.	Dinoflagellate		2.75	0.8	75–126			
*Chlamydomonas reinhardtii*	Freshwater chlorophyte	1050 ppm CO_2_	1.5	1	1000	Photosynthetic O2 evolution and consumption, chla, cell size	Opposite direction from plastic response for cell size, variable for photosynthesis and chla	Collins and Bell (2004)
*Chlamydomonas reinhardtii*	Freshwater chlorophyte	Extremely high (1%) CO_2_	Approximately × 1.15 (average of 3 genotypes)	1 (1 genotype); 1.13 (2 genotypes)	320	n/a		Collins (2011a,b)
*Synechococcus leopoliensis*	Freshwater cyanobacteria	1000 ppmCO2	1.15	0.95	Approximately × 400	n/a		Low-Décarie et al. (2011) nb: plastic and evolutionary responses in for this study are calculated for/relative to ancestor
*Anabaena variabilis*	Freshwater cyanobacteria		1.15	Extinct by end of experiment (?)				
*Navicula pelliculosa*	Freshwater diatom		1.1	1.1				
*Nitzschia palea*	Freshwater diatom		1.1	0.9				
*Pseudokichneriella subcapitata*	Freshwater chlorophyte		1.12	1.05				
*Scenedesmus acutus*	Freshwater chlorophyte		1.1	1.05				
*Cylindrotheca fusiformis*	Diatom	High pCO2 (approx 560 µatm pCO2), High temperature (ambient +5C), High pCO2 and high temperature	1	1.09	185–21	n/a	n/a	Tatters et al. (2013a,b)
*Coscinodiscus sp*.	Diatom		0.84	0.9	169–229			
*Thalassiosira sp*.	Diatom		1	1	179–200			
*Pseudo-nitzschia delicatissima*	Diatom		1	1.13	178–221			
*Navilcula sp*.	Diatom		1	1	188–212			
*Chaetoceros criophilus*	Diatom		1	0.86	194–236			

Chla, chlorophyll a per cell; POC, particulate organic carbon; PIC, particulate inorganic carbon; PON, particulate organic nitrogen.

### Characterizing responses to elevated pCO_2_

To date, few studies have been conducted using experimental evolution to investigate the responses of individual lineages of marine phytoplankton to global change. However, new studies are appearing rapidly, and results are remarkably consistent. A comparison of plastic and evolutionary responses to ocean acidification in different phytoplankton taxa is given in Table 1. In taxa where ocean acidification conditions cause an initial drop in fitness (i.e. growth rate), adaptive evolution occurs. This is the case with coccolithophores, where studies suggest that natural selection partially restores fitness loss by sorting standing variation between clonal lineages, and by heritable change within clonal lineages attributed to novel mutations, since populations reproduce asexually and are founded from single cells, with the caveat that epigenetic effects cannot be ruled out (Lohbeck et al. 2012a; Jin et al. 2013). Calcification, which is thought to be a fitness-related trait in calcifying coccolithophores, can be partially restored over several hundred generations of selection (Lohbeck et al. 2012a; Benner et al. 2013). Although Benner et al. (2013) co-varied higher pCO_2_ levels with higher temperature, hence cause–effect relationships could not be clearly identified, it is interesting that changes in calcification were not associated with changes in expression levels of calcification-related genes. Instead, consistently up-regulated transcripts across several replicate populations were associated with cellular processes and signalling, information storage and processing, metabolism and even viral processes (Benner et al. 2013). A previous experiment where coccolithophore populations evolved under high pCO_2_ conditions also showed phenotypic evidence of functional genetic divergence between replicate populations selected in high pCO_2_ environments, highlighting that there are several possible genetic changes that can be selected during adaptation to ocean acidification conditions, leading to several possible high-CO_2_-adapted genotypes rather than a single repeatable high-CO_2_-adapted genotype (Lohbeck et al. 2012b).

Unlike coccolithophores, who experience a drop in fitness under ocean acidification conditions, other taxa measured thus far are initially insensitive or increase their growth rates in response to CO_2_ enrichment. This includes diatoms, dinoflagellates and chlorophytes (see Table 1 and references therein). In these cases, little if any adaptation occurs in response to ecologically relevant levels of CO_2_ enrichment. This appears consistent with results from studies using freshwater phytoplankton (Low-Décarie et al. 2013), although differences in carbonate chemistry between the high and control CO_2_ treatments may not have been large or consistent enough to drive evolution in the latter study. Rapid evolution has been observed in green algae as well as some diatoms and dinoflagellates (see Table 1). In these cases, evolution did not appear to be adaptive, though there may have been scope for adaptive evolution if experiments had been longer or started with higher standing genetic variation in fitness. In the case of chlorophytes, cell division rates of populations grown at elevated pCO_2_ for hundreds of generations do not increase (and can even decrease) relative to the plastic response of control populations to increases in pCO_2_ (Collins and Bell 2004). This response is repeatable, suggesting that chlorophytes are unlikely to evolve growth rates faster than naïve populations responding plastically to high pCO_2_.

Based on studies to date (see Table 1), some general trends in phenotypic evolution are that first, phytoplankton cells evolved in high CO_2_ tend to be smaller than cells evolved under ambient CO_2_ conditions when placed in high CO_2_. This contrasts with the physiological response to CO_2_ enrichment in chlorophytes and coccolithophores, which is to increase cell size (see Table 1). This is consistent with counter-gradient selection (Conover and Schultz 1995), though it is unclear whether selection acts directly on cell size. Second, high-CO_2_ evolved cells tend to have higher C:N ratios than expected based on the plastic responses of control cells to CO_2_ enrichment, which is in the same direction as the physiological response (Burkhardt et al. 1999; Riebesell et al. 2007). These changes in size and composition could have effects on sinking rates, as well as the food quality of phytoplankton, and has the potential to affect both the composition of marine communities as well as nutrient cycling in the ocean. Finally, at least in chlorophytes, correlations between growth rate and fitness-related traits such as photosynthesis rates can change in either sign, magnitude or both (Collins and Bell 2004). While only one study to date (Benner et al. 2013) characterizes the genetic changes underlying the evolutionary ones, the evolved phenotypes are usually verified using reciprocal transplants (explained in Merilä and Hendry, this issue). In these cases, the phenotypes cannot be explained by plastic responses alone. The use of evolving control populations demonstrates that the evolved phenotypes in these studies are an evolutionary response to the high pCO_2_ environment, rather than to the general lab environment. While information on genetic change provides mechanistic clues for how evolution happens in these cases (Benner et al. 2013), we argue that the question of whether elevated pCO_2_ can drive evolution in marine phytoplankton, and how evolutionary and plastic responses differ, can be answered using standard experimental evolution studies that rely on reciprocal transplants to measure evolutionary responses.

One hallmark of microbial evolution experiments is that they simplify natural systems to the point where individual mechanisms of evolution can be understood. In addition to determining whether and how particular phytoplankton lineages might evolve as a direct response to elevated pCO_2_, several studies have examined whether indirect effects, namely interactions between phytoplankton, are expected to evolve. In particular, can changes in competitive interactions between phytoplankton be predicted from evolutionary changes to the individual lineages? In one study using freshwater communities made up of different taxa (cyanobacteria, chlorophytes and diatoms), individual responses to selection were good indicators of competitive ability and in turn, pairwise competitions were good predictors of shifts in larger community structure (Low-Décarie et al. 2011). However, in three studies using more closely related lineages of either a freshwater chlorophyte or different species of marine diatoms or dinoflagellates, the performance of lineages evolved in isolation was a poor predictor of competitive ability, with lineages that showed changes in growth rate consistent with adaptive evolution having no competitive advantage (Collins 2011a; Tatters et al. 2013a,b). The discrepancy between these studies and the one focused on distantly related taxa (Low-Décarie et al. 2011) may be because closely related lineages or species have similar evolutionary potential and constraints, while distantly related taxa are unlikely to overcome fundamental differences in biology, for example, associated to traits like calcification or N_2_ fixation, during microevolution experiments.

## Plastic responses

Experimental evolution evaluates the potential for evolution in marine phytoplankton. In addition, investigating the metabolic basis of plastic responses could provide clues as to how different taxa might respond to natural selection, especially since functional groups are not closely related, and often have gross differences in biology.

Plastic responses can be measured at the level of the whole cell, and may be an important component of evolutionary response to global change. Here, we define plastic responses as changes in phenotype without any underlying change in genotype. This is in contrast to evolutionary responses, which, by definition, involve a change in mean phenotype of a population due to changes in the genetic composition of that population over time. The connection between plastic and evolutionary responses is discussed immediately following this section. Here, we highlight research aimed at better understanding the plastic responses themselves with the perspective that an understanding of subcellular processes may indicate which processes are likely to be acted on by natural selection. For example, in cases where gross photosynthesis was often not affected by ocean acidification, processes like calcification or N_2_ fixation responded strongly (Kranz et al. 2010; Rokitta and Rost 2012). Process understanding also helps identify environmental changes that are likely to act as evolutionary drivers directly, which can inform the design of evolution experiments. As stated earlier in this issue by Merilä and Hendry, climate change is not only complex but also correlated with many other environmental changes, so that establishing the direct causes of evolutionary change can be difficult. While the authors suggest that experimental evolution is one valid way to isolate the role of particular drivers, limited time and resources dictate that there be some *a priori* reason for carrying out selection experiments to test particular potential drivers and to focus on specific traits.

One example where process studies have shown why different species of marine phytoplankton are more or less responsive to ocean acidification are investigations of carbon concentrating mechanisms (CCMs) (Rost et al. 2003; Kranz et al. 2009). To acquire sufficient carbon for growth, phytoplankton cells must invest resources into CCMs to compensate for the low catalytic efficiency of their CO_2_-fixing enzyme RubisCO. RubisCO limits carbon acquisition due to its low affinity and turnover rate for CO_2_ and its susceptibility to a competing reaction with O_2_ (Badger et al. 1998). The kinetic parameters of RubisCOs differ among phytoplankton taxa, suggesting that some groups should be more prone to carbon limitation than others (Tortell et al. 2000). Phytoplankton has evolved different types of CCMs, and some species can reduce their investment in CCMs under ocean acidification to save energy, which could then be allocated to other processes (Reinfelder 2011). These effects appear strongest in taxa with RubisCOs with poor kinetics – and as a consequence very costly CCMs. Here, process understanding helps to explain, for example, why the cyanobacterium *Trichodesmium* benefits so strongly from ocean acidification, including responses in pathways like N_2_ fixation that are not directly affected by pH or CO_2_ (Kranz et al. 2009, 2010). Diatoms, on the other hand, possess RubisCOs with relatively high CO_2_ affinities, which explain why they show relatively small responses to ocean acidification (Trimborn et al. 2009; Reinfelder 2012). These differences may partially explain the species-specific responses to ocean acidification in growth or elemental composition, and provide testable hypotheses on how and why the action of natural selection could differ among taxa.

### Plastic responses in complex environments

One of the limits of experimental evolution is that ecology is necessarily simplified in experiments designed to understand evolutionary mechanisms or determine whether specific environmental changes can act as evolutionary drivers, so that data on evolutionary responses in marine phytoplankton in response to even the simplest ocean acidification scenarios is sparse. In contrast, there is a much larger and more detailed body of data on plastic responses to ocean acidification, which could be leveraged to predict (or constrain the range of possibilities of) evolutionary responses if plastic and evolutionary responses can be systematically linked. Here, we give a few examples where data on plastic responses to more realistic ocean acidification scenarios exists, and then outline the existing theoretical and empirical studies linking plastic and evolutionary responses.

Currently, little is known about the impact of the combined effects of environmental changes (e.g. ocean acidification plus changes in light intensity, nutrient concentrations and water temperature) on phytoplankton physiology (Rost et al. 2008; Steinacher et al. 2010; Winder and Sommer 2012), but both synergistic and antagonistic interactions between effects have been reported. For example, light intensity appears to influence the beneficial effects of ocean acidification on N_2_ fixation in cyanobacteria and its detrimental effects on calcification in coccolithophores (Kranz et al. 2010; Rokitta and Rost 2012). Likewise, in nutrient-replete conditions, increasing CO_2_ stimulated primary productivity in some phytoplankton species, while under nutrient limitation, increasing CO_2_ did not alter rates of production (Fu et al. 2010; Hoppe et al. 2013).

In addition to having many factors that change simultaneously, natural environments are dynamic. Physiological responses of phytoplankton are typically assessed under quasi-constant environmental conditions using dilute batch or chemostat cultures. For example, light conditions are typically held constant in laboratory experiments but are never constant in the real world – they vary with weather conditions and mixing regime (MacIntyre et al. 2000). This requires that organisms photoacclimate, which may in turn have a cost (MacIntyre et al. 2000; Rost et al. 2006). Nutrient levels and carbonate chemistry are also dynamic, especially over the course of phytoplankton blooms (Arrigo et al. 1999). In addition, fluctuations in pH and CO_2_ will be much larger in the future due to the lowered CO_2_ buffer capacity (Egleston et al. 2010). It has been suggested that more variable environments may select for types with better regulation, and that this may alter the sensitivity of key phytoplankton species to environmental changes (Flynn et al. 2012). A large body of empirical and theoretical literature on evolution in fluctuating environments exists, including complications arising from plastic responses or extreme environmental fluctuations (Lande 2009; Cooper and Lenski 2010; Bonduriansky et al. 2012), though it is rarely applied to the particular case of marine phytoplankton and global change.

Laboratory studies typically use single strains or species. Organismal responses in these cases necessarily lack effects from ecological interactions, such as competition for nutrients, grazing and viral attack. There are some indications that, because of this, predictions of physiological responses of phytoplankton to ocean acidification may underestimate the responses that occur in natural environments. For instance, the absence of CO_2_-dependent changes in growth of many diatom species in laboratory experiments (Reinfelder 2012) would suggest only minor changes in community composition due to ocean acidification. In contrast, pCO_2_ manipulations of natural communities caused significant floristic shifts, particularly in diatom-dominated assemblages (Tortell et al. 2008; Hoppe et al. 2013). For coccolithophores, the ocean acidification-dependent decrease in calcification is far more pronounced in natural assemblages than in single species incubations (Beaufort et al. 2011), since rising CO_2_ causes both reduced calcification rates per cell, and also favours shifts in community composition from more to less calcified species and morphotypes. Hence, alterations in physiological processes affect the mean phenotype both through plastic responses and by changing the species composition of phytoplankton communities.

### Connecting plasticity and evolution

There is significant intraspecific variation in plastic responses, so that lineage sorting is expected to occur under ocean acidification. In the green alga *Ostreococcus tauri*, the plastic responses among 16 strains to manipulated pCO_2_ concentrations were as large as the variation in plastic responses observed between genera (Schaum et al. 2013). Since all strains responded to increased pCO_2_ with higher rates of growth and photosynthesis, the species *O. tauri* will likely benefit from ocean acidification with the more plastic strains increasing in their relative abundance. Similarly, in the common and widespread coccolithophore *E. huxleyi*, strain-specific differences in ocean acidification-responses have been observed (Langer et al. 2009; Hoppe et al. 2011), which may lead to an intraspecific shift from more sensitive to less sensitive strains (Beaufort et al. 2011). Variation in plastic responses to ocean acidification has also been seen in diatoms and dinoflagellates (Kremp et al. 2012). This variation in plastic responses within species strongly suggests that there is variation in evolutionary potential within species as well, and that it is important to take intraspecific diversity into account both in physiological and evolutionary studies aimed at understanding natural populations.

Theoretical models predict that phenotypic plasticity has the potential to affect whether or not populations adapt in suboptimal environments, as well as the rate and direction of evolution. For example, higher levels of adaptive plasticity should increase the probability of populations persisting in the face of environmental deterioration, partly by keeping population size large enough that adaptation is possible (Lande 2009; Chevin et al. 2010). In addition, phenotypic plasticity is predicted to facilitate evolution and affect phenotypic outcomes because plastic traits should express greater mutational variance, have higher standing genetic variation, and be more evolvable (Draghi and Whitlock 2012). However, adaptive plasticity has also the potential to shield genotypes from natural selection if optimal phenotypes can be produced by plasticity alone, at least in the absence of a cost of plasticity (for a review of how different types of plasticity may affect adaptive evolution, see Ghalambor et al. (2007)). To date, there are no direct empirical tests of how plasticity and evolution are related for large populations of phytoplankton.

## Standing genetic variation

### Clonal diversity in field populations of marine phytoplankton

The number of distinct clonal lineages, also termed clonal diversity (Ellstrand and Roose 1987), in a population is an important measure of standing genetic variation because it both is readily available to lineage sorting (Becks et al. 2010) and partially determines the potential magnitude and rate of response to selection. Until recently, clonal diversity could not be measured directly and several hypotheses existed on how much might be found in field populations. On one hand, the rapid rate of asexual reproduction led some to suggest that clonal diversity would be very low due to past, strong selective sweeps of the population, and low clonal diversity was indeed observed using early protein-based methods of assessing variation (e.g. Gallagher 1980). On the other hand, the enormous census sizes of these organisms (thousands to millions of cells per litre), in addition to extensive physiological diversity observed (Carpenter and Guillard 1971; Brand 1981; Gallagher 1982) led others to suggest that clonal diversity could be quite high (Doyle 1975; Brand 1990).

Recent studies using genetic markers such as microsatellites show that there is high standing genetic variation in natural phytoplankton populations. High diversity (80–100% unique genotypes) has been found in every phytoplankton taxon examined, including diatoms, coccolithophores and dinoflagellates (Table 2 and references therein). Sample sizes usually range from 10 to 30 individuals per population, making estimates of clonal population size rare. One exception is the marine diatom *Ditylum brightwellii*. Over 600 individuals were genotyped during a spring bloom, and it was estimated that the population comprised at least 2400 different genotypes (Rynearson and Armbrust 2005). As expected, census sizes were much higher, reaching >10 000 cells/L. Aside from this example, there are no other estimates of clonal population size in diatoms or any other taxa of marine phytoplankton.

**Table 2:** Gene and clonal diversity among phytoplankton taxa. Gene diversity is reported as expected heterozygosity (*H*_e_) and clonal diversity as the ratio of the number of unique genotypes (G) to the total number of isolates examined (N). In cases where only one population was analysed, the average *H*_e_ and standard deviation over all loci are listed

Organism	Gene diversity (*H*_e_)	Clonal diversity (G:N)	References
Coccolithophores			
*Emiliania huxleyii*	0.72–0.78	1.00	Iglesias-Rodriguez et al. (2006)
Diatoms			
*Ditylum brightwellii*	0.70–0.88	0.87–0.99	Rynearson and Armbrust (2000, 2004, 2005), Rynearson et al. (2006)
*Pseudo-nitzschia multiseries*	0.39–0.70	0.92	Evans et al. (2004)
*Pseudo-nitzschia pungens*	0.53–0.83	0.95–0.98	Evans et al. (2005), Casteleyn et al. (2009, 2010)
*Skeletonema marinoi*	0.56–0.71	0.99–1.00	Godhe and Härnström (2010), Härnström et al. (2011)
Dinoflagellates			
*Akashiwo sanguinea*	0.56 ± 0.26	NA	Cho et al. (2009)
*Alexandrium fundyense*	0.54 ± 0.13	0.47–0.97	Erdner et al. (2011), Richlen et al. (2012)
*Alexandrium minutum*	0.61–0.88	NA	McCauley et al. (2009)
*Alexandrium tamarense*	0.62–0.77	1.0	Nagai et al. (2007), Alpermann et al. (2010)
*Cochlodinium polykrikoides*	0.54 ± 0.21	NA	Nagai et al. (2009)
*Oxyrrhis marina*	0.61–0.72	0.92	Lowe et al. (2010)
Raphidophytes			
*Heterosigma akashiwo*	0.65 ± 0.17	NA	Nagai et al. (2006)

NA indicates not available.

**Figure 2:**
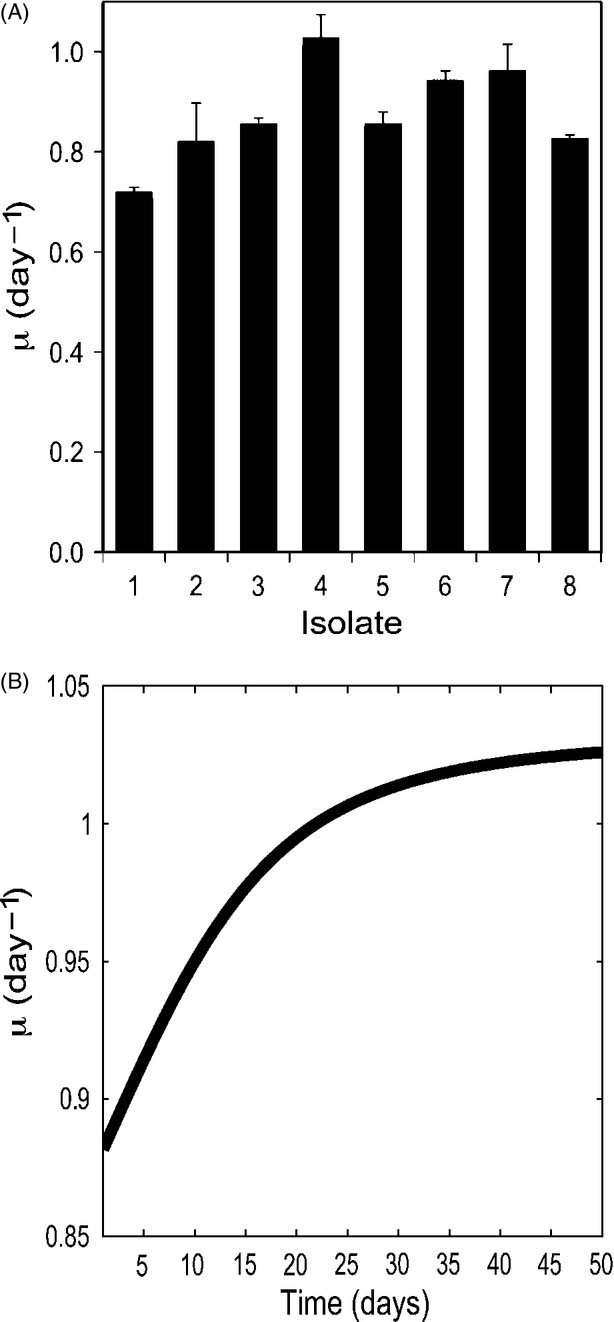
(A) Intraspecific variation in growth rate (μ) among eight isolates of the diatom *Ditylum brightwellii* collected from Hood Canal, WA, USA (Adapted from Rynearson and Armbrust 2000). B) Simulation of the change in population growth rate (μ) over time. At time zero, all eight isolates represent an equal fraction of the population and thus the population growth rate is an average of the individual growth rates in panel A. Over time, the fastest-growing isolates become more abundant in the simulated population, driving average population growth rates up.

In the few cases where it has been measured, high levels of clonal diversity appear to be matched with significant variation in fitness or traits correlated with fitness (Rynearson and Armbrust 2000, 2004; Schaum et al. 2013). Although in nearly all cases, the important adaptive traits for phytoplankton are unknown. However, high levels of clonal diversity suggest that these rapidly dividing organisms may be able to respond quickly to climate change through selection acting on standing genetic variation. For example, light intensity regulates phytoplankton growth rates and is expected to change in response to a warming ocean (Winder and Sommer 2012). A combined laboratory and modelling study of the effect of light intensity on eight diatom genotypes with different growth rates predicted that selection could dramatically shift the genotypic composition of a population within 14 days (Rynearson and Armbrust 2000), and could change population growth rates (Fig. 2). Similarly, a recent laboratory study revealed significant selection in initially multiclonal populations in response to low pH after 160 days, with the lowest pH leading to the most dramatic reduction in clonal diversity (Lohbeck et al. 2012a). In both cases, populations adapted by sorting, showing reduced clonal diversity, and an increase in the mean fitness of the population, as expected under natural selection. These two studies are consistent, but both used few clones (<10) and so may not reflect the very high standing diversities in natural populations. High standing diversity may slow evolution through clonal interference, where beneficial genotypes compete with each other and variation is purged slowly (Gerrish and Lenski 1998), or it may speed evolution through sign epistasis, where beneficial alleles can occur on multiple genetic backgrounds, which can alter the fitness effect of the allele (Hayden et al. 2011). In any case, additional laboratory, modelling and field studies are needed to better understand the rate and magnitude of genotypic selection in response to climate change factors.

### Gene diversity in marine phytoplankton

Since significant genetic diversity can be released into a population following sexual reproduction, a second measure of standing genetic variation is gene diversity. Gene diversity or expected heterozygosity (see Box 1) has been generally high in all phytoplankton examined thus far. Using microsatellite markers, gene diversity ranged from 0.72 to 0.78 in coccolithophores, from 0.39 to 0.88 in diatoms from 0.54 to 0.88 in dinoflagellates and was 0.65 ± 0.17 in the raphidophyte *Heterosigma akashiwo* (See Table 2 for references). These measures using microsatellite markers are supported by whole genome sequences. For example, a nucleotide polymorphism of 0.75% was observed between homologous chromosomes in the diploid diatom *Thalassiosira pseudonana* (Armbrust et al. 2004) and about 1% among strains of the haploid chlorophyte *Ostreococcus tauri* (Grimsley et al. 2010), which are comparable to levels previously observed in marine animals (Aparicio et al. 2002; Dehal et al. 2002).

Like clonal diversity, the high levels of gene diversity within and among individuals in nearly all taxa examined suggest that there is sufficient diversity in field populations for them to evolve or adapt in response to global change. For example, ocean temperatures are warming in response to increased air temperatures (Meehl et al. 2007), generating a potentially large selection pressure. Interestingly, positive selection in a suite of genes has been observed among multiple genomes of *T. pseudonana* collected from locations with different annual temperature fluctuations (Koester et al. 2013). Furthermore, intraspecific genetic variation in a range of phytoplankton species appears highest at the extremes of temperature tolerance, further suggesting that selection could act efficiently as water temperatures increase (Boyd et al. 2013). These studies suggest that the potential for an evolutionary response to changes in climate is high. In field populations, the high potential for evolutionary change conferred by high genetic diversity will likely be balanced by factors constraining evolutionary responses including clonal interference, large population sizes and infrequent sexual reproduction (see below).

### Local adaptation

The high levels of gene and clonal diversity observed in marine phytoplankton are often subdivided into genetically distinct populations. This is surprising because phytoplankton drift passively with tides and currents, so that they appear to have enormous potential for long-range and persistent dispersal along with high levels of gene flow between populations. Despite this potential, population subdivision has been reported in dinoflagellates, diatoms and coccolithophores. In some cases, genetic divergence is related to increasing geographic distance, as in the diatom *Pseudo-nitzschia pungens* (Casteleyn et al. 2010) and the coccolithophore *E. huxleyi* (Iglesias-Rodriguez et al. 2006). In others, genetically distinct populations exist in close proximity to one another, as in the dinoflagellate *Alexandrium fundyense* (Richlen et al. 2012) and the diatom *Ditylum brightwellii* (Rynearson and Armbrust 2004) suggesting that distance is not the main driver of genetic differentiation. In cases where distance does not explain divergence, it has been suggested that circulation patterns, such as recirculation in a coastal fjord, may provide a sufficient barrier to gene flow, allowing populations to diverge and perhaps adapt to local conditions (Rynearson and Armbrust 2004). Combined with the high standing genetic variation in natural populations, this suggests that the potential to adapt to changing conditions is present in marine phytoplankton, although the relative importance of adaptation by local populations versus replacement by immigrant types has yet to be established.

**Box 1**

The marine and evolutionary literature use different vocabulary (in some cases to describe the same thing). In this review, we have used the conventions of the primary literature for each section. The definitions here are used for microbial studies, and may not translate to studies on multicellular or obligately sexual organisms.

**Adaptive evolution:** Evolution where fitness increases as a result of natural selection acting. Alternately, evolution may be neutral (no change in fitness) or maladaptive (decrease in fitness). Note that many of the marine studies cited are interested a fitness-related trait (such as calcification) rather than fitness itself, and tend to frame results around trait evolution.

**Clonal diversity:** The number of different genotypes that exist within a population. In the absence of mutation and sexual recombination, this is the amount of genetic variation that natural selection has to act on.

**Evolutionary change/response:** Change in genotype frequency within a population between generations. Genetic variation can originate from *de novo* mutation, or may already be present as standing variation. Heritable changes in fitness are often used in place of measuring genotype frequencies directly in microbial experiments. For simplicity in this review, we use ‘changes in genotype’, but are aware that this may include non-genetic contributions such as epigenetic effects where changes in genotype are not measured directly.

**Evolution within lineages:** Changes in genetic composition within lineages where mutation is the source of variation.

**Fitness:** The average reproductive success of a genotype in a particular environment (Elena and Lenski 2003). In practice, experimental evolution uses either growth rate or competitive ability as a measure of fitness for selection experiments carried out in semi-continuous (batch) culture, or population carrying capacity in continuous culture (chemostat).

**Gene diversity/ expected heterozygosity:** The probability that, at a single locus, any two alleles chosen at random from the population will be different from each other. The average gene diversity over many loci is another estimate of the extent of genetic variation within a population.

**Lineages/clonal lineages:** Genotypes within a species. Clonal lineages are founded from a single individual and propagate asexually so that all variation within a lineage over the timescale of interest (e.g. during an experimental evolution study) comes from mutation. Clonal lineages are often referred to as ‘lines’ in experimental evolution studies.

**Plastic response/acclimation response:** A response of individual organisms to environmental change that involves a change in phenotype with no underlying change in genotype. Plasticity is adaptive when it increases fitness.

**Sorting between lineages/lineage sorting:** Also referred to as ‘sorting standing genetic variation.’ Changes in the genetic composition of a population where more fit lineages in a population become more frequent and less fit lineages becoming less frequent as a result of natural selection. In this case, the different lineages are present at the onset of the time window of interest rather than generated by mutation during the experiment or time of interest.

## Conclusions and future directions

Do marine phytoplankton evolve in response to global change, and if so, how? The simplest interpretation of laboratory selection experiments to date is that most taxa can evolve by genetic change within species, using standing genetic variation, mutation or both. In cases where growth under ocean acidification conditions decreases fitness, it can be at least partially restored through natural selection – these populations adapt, though it is unclear whether fitness can be completely restored. In natural populations, the high standing genetic variation further suggests a high potential for evolutionary responses to climate change.

Before that simple interpretation can be evaluated, several considerations (besides simple lack of data) must be dealt with. First, laboratory studies simplify both the abiotic and biotic features of natural environments. Many of the studies cited in this review change only a single environmental factor (usually pCO_2_), but many simultaneous environmental changes are occurring in the oceans. While current evolution experiments suggest that some taxa can evolve in response to ocean acidification, genetic constraints are less likely to hinder evolution in simple laboratory environments where only few traits are under selection. In natural field populations, many traits are under selection and genetic correlations among them may limit or constrain adaptation when multiple environmental changes occur. As a result, laboratory experiments may underestimate the strength of constraints on evolutionary responses to global change. Second, demography and life history will affect the strength and effectiveness of natural selection. This includes clonal interference during blooms consisting of thousands of clonal lineages, recombination rates ranging from 2 to 40 years, the formation of resting spores with long-term storage potential, and multiple ploidy levels, even within individual species. Currently, there is little information on the demography and life history of marine phytoplankton that would allow us to take these effects into account. Third, ecological interactions such as competition or top-down effects will also impose selection, and are likely to be affected by global change, yet few evolutionary studies include more than one trophic level. Because of this, gathering basic life history, demographic and ecological data in a context that is useful for evolutionary inference is essential.

One area where data are plentiful is on plastic responses of several key species of marine phytoplankton to aspects of global change. One challenge for interpreting the existing literature is that most often, single strains have been used when comparing taxa. Given the knowledge that there is considerable intraspecific variation in plastic responses, it is prudent that future studies incorporate both intra- and interspecific variation. Alongside this, there is a body of theoretical literature on how and why plastic responses should affect evolutionary ones. Here, marine phytoplankton offer excellent model systems in which to test and expand this body of theory in ecologically relevant systems while leveraging the data on plastic responses to tell us about evolution.

Every indication so far suggests that marine phytoplankton have the potential to evolve in response to global change, both by sorting standing variation in fitness and by using *de novo* mutation. The statement that large phytoplankton populations can evolve on timescales of years or decades is not surprising – the interesting question about marine phytoplankton evolution under global change is whether and how this will affect the ecology and biogeochemistry of the world's oceans. Our review has highlighted studies that examine plasticity or evolution in very simplified laboratory systems, or that use genetic tools in natural populations. Unlike other microbial model systems for experimental evolution, our main motivation for studying marine phytoplankton is their ecological and biogeochemical importance. While theory and laboratory experiments tell us what can happen and why, studying natural populations tells us what does happen in the real world. It is now vital to systematically link responses seen in laboratory studies to changes in natural populations so that we can detect and understand changes to marine phytoplankton populations in changing oceans.
